# Tissue Adhesive, Conductive, and Injectable Cellulose Hydrogel Ink for On-Skin Direct Writing of Electronics

**DOI:** 10.3390/gels8060336

**Published:** 2022-05-30

**Authors:** Subin Jin, Yewon Kim, Donghee Son, Mikyung Shin

**Affiliations:** 1 Department of Intelligent Precision Healthcare Convergence, Sungkyunkwan University (SKKU), Suwon 16419, Korea; subinjin@g.skku.edu; 2 Department of Electrical and Computer Engineering, Sungkyunkwan University (SKKU), Suwon 16419, Korea; ywkim0726@gmail.com; 3 Department of Superintelligence Engineering, Sungkyunkwan University (SKKU), Suwon 16419, Korea; 4 Center for Neuroscience Imaging Research, Institute for Basic Science (IBS), Suwon 16419, Korea; 5 Department of Biomedical Engineering, Sungkyunkwan University (SKKU), Suwon 16419, Korea

**Keywords:** carboxymethylcellulose, tannic acid, conductive hydrogel, injectable hydrogel, adhesive hydrogel, 3D printing, direct printing

## Abstract

Flexible and soft bioelectronics used on skin tissue have attracted attention for the monitoring of human health. In addition to typical metal-based rigid electronics, soft polymeric materials, particularly conductive hydrogels, have been actively developed to fabricate biocompatible electrical circuits with a mechanical modulus similar to biological tissues. Although such conductive hydrogels can be wearable or implantable in vivo without any tissue damage, there are still challenges to directly writing complex circuits on the skin due to its low tissue adhesion and heterogeneous mechanical properties. Herein, we report cellulose-based conductive hydrogel inks exhibiting strong tissue adhesion and injectability for further on-skin direct printing. The hydrogels consisting of carboxymethyl cellulose, tannic acid, and metal ions (e.g., HAuCl_4_) were crosslinked via multiple hydrogen bonds between the cellulose backbone and tannic acid and metal-phenol coordinate network. Owing to this reversible non-covalent crosslinking, the hydrogels showed self-healing properties and reversible conductivity under cyclic strain from 0 to 400%, as well as printability on the skin tissue. In particular, the on-skin electronic circuit printed using the hydrogel ink maintained a continuous electrical flow under skin deformation, such as bending and twisting, and at high relative humidity of 90%. These printable and conductive hydrogels are promising for implementing structurally complicated bioelectronics and wearable textiles.

## 1. Introduction

Flexible and soft electronics, such as wearable monitoring devices [1,2,3], sensors [4,5,6,7], electronic skin [8,9], and human–machine interfaces [10], have recently attracted attention in the biomedical field [11,12,13]. Owing to their deformable characteristics, flexible electronics can be used as next-generation electronic devices that contact the human skin instead of conventional hard electronic devices [14,15]. In recent studies, a strategy for printing inexpensive and flexible electronic materials called electronic tattoos or epidermal electronics to directly write on the skin was evaluated [16,17]. Printed electronics on the skin can be used in various fields, such as light-emitting diodes (LEDs) [18], sensors [19,20], and transistors [21]. To achieve a high conductivity efficiency in printed electronics, most studies have focused on the low resistance, stable mechanical properties, and printability of conductive ink [22,23,24]. However, few studies have been conducted on maintaining its stability on elastic skin tissues, and synthetic polymers have been used to maintain electronic circuits and variable physical properties.

The application of direct writing to sensitive biological tissues requires high levels of flexibility, repetitive deformation due to movement, and low toxicity [25,26]. Hydrogels are useful for satisfying these conditions because of their tissue-like mechanical properties, conductivity, and biocompatibility [27,28,29]. In particular, cellulose, which is found in many plants, has been used for flexible/transparent substrates, separators, electron and ion conductors, electrolytes, and electrodes owing to its flexibility, ease of fabrication, mechanical strength, and biodegradability [30,31,32,33]. Cellulose can also be used as an ink for direct writing because of its stable mechanical properties for deformation, injectability, and biocompatibility [34,35,36]. However, natural polymer-based hydrogels are unsuitable for printing on tissue systems because of their low tissue adhesion strength and injectability due to covalent crosslinking [37,38]. To resolve these issues, polyphenol is a good candidate for providing tissue adhesion capability and injectability to natural polymer-based hydrogel inks. Polyphenols, which have a large number of phenol groups derived from various plants, may exhibit self-healing and injectable properties through stable structure maintenance of the hydrogel and reversible crosslinking through many hydrogen bonds with the polymer backbone, as well as π–π stacking of the polyphenol itself [39,40]. In addition, because polyphenols are known to have strong adhesion to tissues, they are often used to manufacture adhesive ink [38,41]. In particular, tannic acid (TA) is used to produce a conductive hydrogel with metal ions by forming a metal–phenolic network with variable metal ions and may exhibit strong adhesion to a structure [42,43].

**Figure 1:**
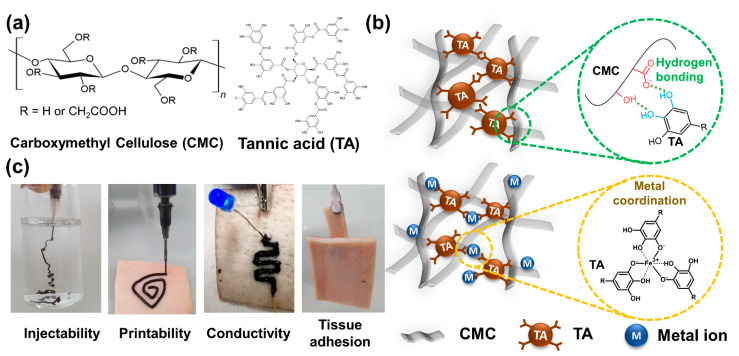
Schematic of the conductive adhesive CMC hydrogel. (**a**) Chemical structures of CMC and TA. (**b**) Fabrication mechanisms for the AC and CAC hydrogels. (**c**) Digital images of various properties of the CAC hydrogel.

In this study, the fabrication of conductive and adhesive cellulose (CAC) hydrogel ink was evaluated by mixing TA with three types of metal ions—gold chloride (HAuCl_4_), silver nitrate (AgNO_3_), and ferric chloride (FeCl_3_)—and carboxymethyl cellulose (CMC) (Figure 1a). The carboxylate group of CMC and the hydroxy group of TA form strong hydrogen bonds. The adhesive cellulose (AC) hydrogel has tissue adhesive properties owing to TA, self-healing properties, and injectability through non-covalent hydrogen bonding (Figure 1b, top). In addition, in CAC hydrogels, metal–carboxyl coordination and metal–phenolic networks are formed when metal ions of Au, Ag, and Fe are mixed together in the AC hydrogel (Figure 1b, bottom) [43,44,45]. The CAC hydrogel prepared as described above has injectability due to non-covalent crosslinking and can be used as an ink for direct printing on tissues through tissue adhesion. It also has conductivity because it contains metal ions (Figure 1c). To demonstrate and optimize the mechanical and electrical properties of the CAC hydrogel, the rheological behavior, adhesiveness, and resistance to various metal ions and concentrations in the hydrogel were evaluated. Based on the investigated characteristics, we successfully demonstrated that the LED maintained stable light emission without electrical malfunction even under various deformations of the electronic circuit printed directly on the tissue.

## 2. Results and Discussion

### 2.1. Fabrication and Rheological Characterization of AC Hydrogels

A common approach for the fabrication of AC hydrogels is the mixing of CMC and TA. High-viscosity hydrogels are manufactured because the carboxyl and hydroxyl groups of CMC form strong hydrogen bonds with the phenol groups of TA. Therefore, the number of functional groups capable of hydrogen bonding depends on the change in the concentration of CMC and TA, and the mechanical properties of the hydrogel are determined. Therefore, hydrogels were prepared with different concentrations of CMC and TA and named CMC_0.5_, AC-1, AC-2, and AC-3 (Table 1).

**Figure 2:**
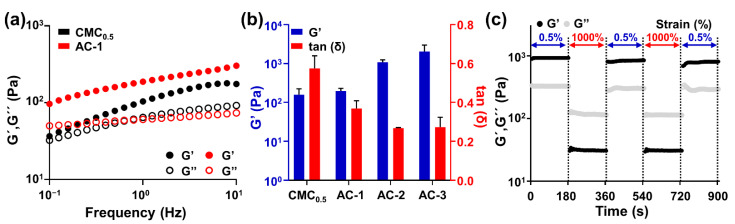
Rheological characterization of the AC hydrogel with different concentrations of CMC. (**a**) Oscillation frequency sweep measurements of CMC_0.5_ (black) and AC-1 (red) hydrogels. The filled circles represent the storage modulus (G′), and the empty circles represent the loss modulus (G″). (**b**) Comparison of the storage modulus and tan(δ) values among CMC_0.5_, AC-1, AC-2, and AC-3. (**c**) Disruption and recovery of the storage (G′) and loss (G″) moduli of AC-3 hydrogels under alternating strains of 0.5% and 1000%.

The rheological properties of the AC hydrogels were evaluated in CMC and TA solutions at different volume ratios (Figure 2a,b). As indicated by the graphs of the storage (G′) and loss (G″) moduli of CMC_0.5_ and AC-1 measured at frequencies ranging from 0 to 10 Hz, the storage modulus increased over all frequency ranges, in addition to TA, indicating that the interaction between TA and CMC created a solid hydrogel (Figure 2a). In addition, measuring the storage modulus and tan(δ) at different volume ratios (AC-1, AC-2, and AC-3) of CMC and TA indicated that the storage modulus increased and tan(δ) decreased as the CMC concentration increased. Therefore, the mechanical properties of the AC hydrogel depended on the concentrations of CMC and TA, and the most stable hydrogel was AC-3. Reversible crosslinking due to hydrogen bonding between TA and CMC resulted in AC hydrogels with self-healing properties, which make them stable during injection, printing, and deformation after printing. To demonstrate this, we investigated the changes in the mechanical modulus of the AC hydrogels upon treatment with urea—which has a role of disrupting multiple hydrogen bonds (Figure S1). If the hydrogen bonds between CMC and TA are major driving forces for the gelation, the mechanical modulus would decrease upon adding urea into the polymeric network. As expected, a decrease in both the G′ and G″ values of the hydrogels was monitored as urea concentration became high, clearly indicating that the hydrogen bonds between CMC and TA are involved in the gelation. In addition, to evaluate the self-healing properties of the AC hydrogels, the storage and loss moduli were measured (Figure 2c). When AC-3 hydrogels were measured at a frequency of 1 Hz with strains of 0.5% and 1000% at 3-min intervals, recovery of the storage modulus to the 0.5% strain level after 1000% strain was observed.

### 2.2. Fabrication and Rheological Characterization of CAC Hydrogel

**Figure 3:**
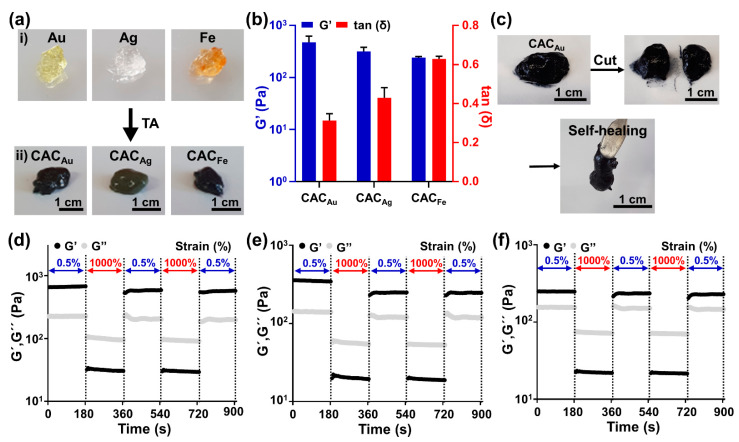
Fabrication and mechanical characterization of CAC hydrogels. (**a**) Digital images of (i) the CMC and metal ion mixture and (ii) CAC_Au_, CAC_Ag_, and CAC_Fe_. (**b**) Comparison of the storage modulus (G′, blue) and tan(δ) (red) values among CAC_Au_, CAC_Ag_, and CAC_Fe_. (**c**) Macroscopic self-healing images of CAC_Au_. (**d**–**f**) Evaluation of the disruption and recovery of the storage (G′) and loss (G″) moduli under alternating strains of 0.5% and 1000%: (**d**) CAC_Au_; (**e**) CAC_Ag_; (**f**) CAC_Fe_.

Various studies have been conducted to form metal–phenolic networks by reacting TA with metal ions to fabricate conductive hydrogels [44,46,47]. In this study, metal–carboxylate coordination and metal–phenolic networks were constructed using Au, Ag, and Fe ions to fabricate conductive hydrogels. When 20 μL of metal ions was mixed with 0.3 mL of CMC, the metal ions and carboxylate of CMC changed to a weak gel through metal coordination bonding (Figure 3a, top). When 0.8 mL of TA was added to the metal ion and CMC mixture, the hydrogel became sticky (Figure 3a, bottom). The color and mechanical properties of the CAC hydrogel, along with the labeling of the samples, depended on the type of metal ion (Table 2). In addition, the rheological properties of the CAC hydrogel depended on the type of metal ion (Figure 3b). There were no significant differences in the storage modulus: 476.9 ± 10.5 Pa for CAC_Au_, 319.1 ± 63.8 Pa for CAC_Ag_, and 242.2 ± 12.6 Pa for CAC_Fe_. However, differences were observed for tan(δ): CAC_Au_ (0.31 ± 0.03), CAC_Ag_ (0.43 ± 0.05), and CAC_Fe_ (0.63 ± 0.01); CAC_Au_ was the toughest, and CAC_Fe_ was the softest. These results indicate that the degree to which TA–TA interactions and CMC–TA interactions interfere depends on the type of metal ion.

The self-healing property of the CAC hydrogel, which is caused by metal ions and phenol groups, suppresses the breakage of the crosslinking by shear stress during printing and the damage to the printed electronics caused by surface deformation after printing. Rheological property analysis, cutting, and contact experiments were conducted to demonstrate the self-healing properties of the CAC hydrogel. When the bulk CAC_Au_ hydrogel was cut in half and brought into contact again, CAC_Au_ was immediately reconstructed (Figure 3c). In addition, in the rheological behavior analysis, the storage modulus, which decreased from the 1000% strain, recovered from the 0.5% strain (Figure 3d). This rheological behavior was also observed for CAC_Ag_ and CAC_Fe_ hydrogels (Figure 3e,f). These results indicate that CAC hydrogels can self-heal with the fast recombination of reversible metal coordination bonds between CMC/TA, and metal ions and hydrogen bonds between CMC and TA.

### 2.3. Tissue Adhesion Capability of CAC Hydrogel

**Figure 4:**
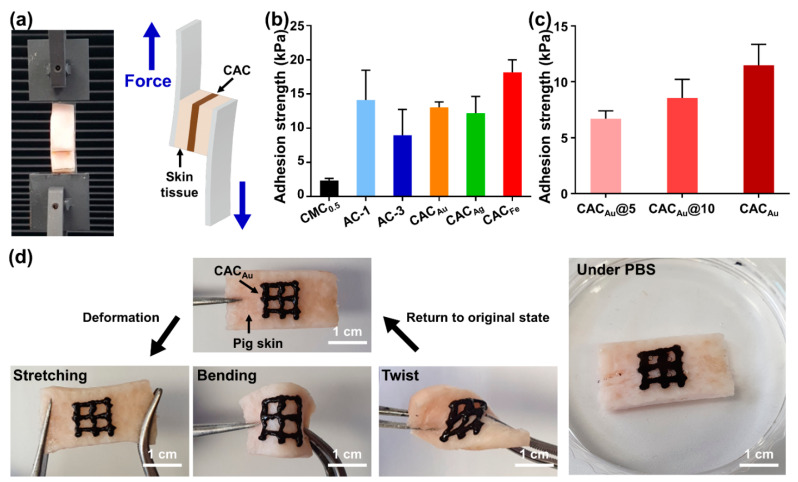
Tissue adhesion capabilities of CAC hydrogels. (**a**) Digital images and schematic of the shear adhesion test method using a UTM. (**b**) Evaluation of the shear adhesion strength of CMC0.5, AC-1, AC-3, CAC_Au_, CAC_Ag_, CAC_Fe_. (**c**) Evaluation of the shear adhesion strength of CAC_Au_ depending on the amount of gold ions. (**d**) Macroscopic images of tissue adhesion in direct writing of CAC_Au_ on tissue with various deformations of pig skin (**left**) and the stability in PBS (**right**).

In the direct printing of electronics on the skin, the tissue adhesion capability of ink is important for the electronics to remain stable on the skin. The strong tissue adhesion of TA in CAC hydrogels allows the direct printing of hydrogel ink. To evaluate the tissue adhesion capability of the CAC hydrogel, shear adhesion tests were conducted on skin tissues using a universal testing machine (UTM) (Figure 4a). The adhesion strength of CMC_0.5_ was 2.3 ± 0.3 kPa, indicating that CMC_0.5_ has non-tissue adhesion properties (Figure 4b). The adhesion strength of AC-1 was 14.1 ± 4.3 kPa, which was higher than that of AC-3 (8.9 ± 3.8 kPa). This was caused by an increase in the tissue adhesion strength due to the larger amount of TA. The adhesion strengths of CAC_Au_ and CAC_Ag_ were 13.0 ± 0.8 and 12.2 ± 2.5 kPa, respectively (similar to that of AC-1), and CAC_Fe_ exhibited the highest adhesion strength among the samples (18.2 ± 1.8 kPa). In addition, the amount of gold ions used in the gel formation was adjusted to 5, 10, and 20 µL, and the resulting adhesion was evaluated (Figure 4c). The tissue adhesion strength increased with the amount of gold ions (from 6.7 ± 0.7 kPa for CAC_Au_@5 to 8.5 ± 1.7 kPa for CAC_Au_@10). The results for the tissue adhesion capability of the CAC hydrogel indicated that the addition of metal ions to the AC hydrogel enhanced the stability of the hydrogel structure through the formation of a metal–phenolic network, which increased the cohesive strength and thus the adhesive strength. Furthermore, to demonstrate that the tissue adhesion capability of CAC hydrogels supports the direct printing of electronics on tissue to maintain a stable contact, a qualitative evaluation was conducted involving the deformation of skin tissue in various forms after direct printing of a CAC hydrogel with a 23-gauge needle on pig skin (Figure 4d). At the maximum tensile, bending, and twist deformations of the pig skin, CAC_Au_ remained stable, without separation from the tissue. In addition, when the pig skin was immersed in a 1× phosphate-buffered saline (PBS) solution for 30 min, the initial form was maintained, without excessive swelling.

### 2.4. Electrical Properties of CAC Hydrogel

**Figure 5:**
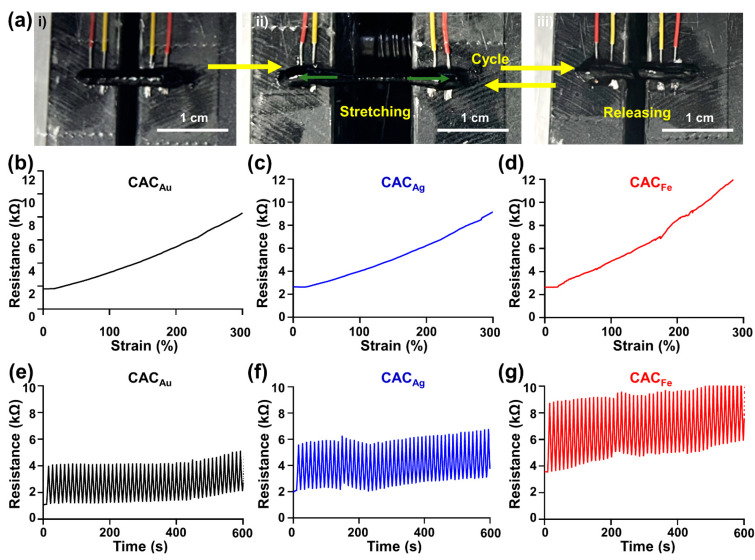
Electrical characterization of CAC hydrogels. (**a**) Macroscopic images: (i) pristine; (ii) stretching; (iii) release. (**b**–**d**) Continuous tensile strain of the samples at a speed of 3 mm/min: (**b**) CAC_Au_; (**c**) CAC_Ag_; (**d**) CAC_Fe_. (**e**,**f**) Repetitive cyclic stretching test with strains ranging from 0% to 200% at a speed of 1 mm/s for 10 min: (**e**) CAC_Au_; (**f**) CAC_Ag_; (**g**) CAC_Fe_.

For long-term use, electronic devices printed on the skin must resist excessive increases in electrical resistance due to the daily movement of the skin. To evaluate the change in resistance per unit tensile strain of the CAC hydrogel, we followed the experimental method used in a previous study (Figure 5a) [48]. Regardless of the type of metal ion, the initial resistance of the CAC hydrogel was maintained at approximately 2 kΩ (Figure 5b–d). According to the results of a tensile strain test, the resistances of CAC_Au_ (Figure 5b) and CAC_Ag_ (Figure 5c) at 300% strain increased to 8351 and 9155 Ω, respectively, while the resistance of CAC_Fe_ (Figure 5d) increased rapidly with 300% strain to 12,617 Ω. In addition, in a 10-min cyclic stretching test (0–200% strain), CAC_Au_ maintained a resistance difference of ≤3 kΩ for the initial resistance, and CAC_Ag_ maintained a resistance difference of ≤4 kΩ (Figure 5e,f). CAC_Fe_ also maintained a constant change in resistance, but the resistance increased to 10 kΩ (Figure 5g). Compared with the CAC hydrogels containing other metal ions, a larger conductivity loss occurred during tensile deformation owing to the strong interaction between TA and Fe ions. Therefore, CAC_Au_ is the best candidate for use in direct tissue writing electronics, and these results provide an appropriate electrical demonstration of the electrical properties of each of the different CAC hydrogels.

### 2.5. Electrical Properties of the Filaments Printed Using CAC Hydrogel Inks

These rheological, adhesive, and electrical properties of the CAC hydrogel inks enable their direct writing on biological tissue. Considering the further biomedical potential of the inks, we confirmed their cytocompatibility at 24 and 48 h after the treatment of the CAC_Au_ ink (Figure S2). As a result, ~90% of the cells were alive upon treatment of the sample below 1 mg/mL. Furthermore, the concentration of TA included in the CAC_Au_ ink was 250 mg/mL, which is less than the TA concentration previously used in the fabrication of tissue adhesive materials implantable in vivo [49,50]. That is, the inks might not cause severe toxicity and skin irritation after direct on-tissue printing.

**Figure 6:**
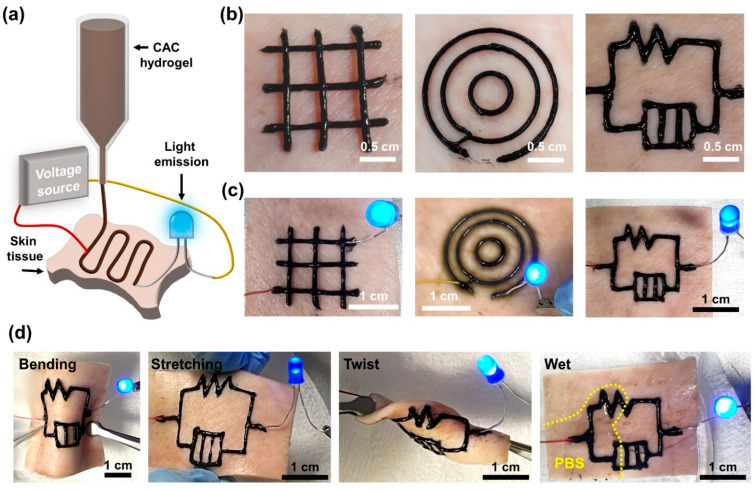
Evaluation of electronics directly printed on tissue with the CAC hydrogel. (**a**) Schematic of the direct-printing process. (**b**) Macroscopic images of concentric (**left**), grid (**middle**), and circuit-shaped (**right**) electronics printed using the CAC_Au_ hydrogel. (**c**) Photographs of LED emission with printed electronics. (**d**) LED light emission with a stable conductivity under stretching (**left**) and bending (**right**) deformation.

To evaluate the electrical properties and conductivity stability of CAC electronics printed directly on skin tissues before and after tissue deformation, CAC_Au_ hydrogels were printed on porcine skin using a three-dimensional (3D) printer (Figure 6a). In addition, CAC_Au_ printed in the form of concentric circles, grids, and circuits on the skin tissue had a space for wire and LEDs to contact. The printed electric circuit consisting of CAC_Au_ had a high resolution and a uniform filament structure, and no slippage from the skin or breakage of ink was observed during the printing process (Figure 6b). Furthermore, in the evaluation of the circuit functionality using LEDs, light emission from both sides was observed when zigzag and concentric circuits were directly printed and connected to LEDs (Figure 6c). In addition, despite deformations such as stretching, bending, and twisting of the skin tissue, the conductivity was maintained, and light emission of the LEDs was observed (Figure 6d). Moreover, when half of the skin tissue with printed electronics was immersed in PBS, the LED was emitted without electrical malfunction. These results indicate that CAC hydrogels are effective for the on-skin direct writing of electronics.

### 2.6. Characterization of CAC Hydrogels under High Humidity Condition and Repeated Tissue Deformation

**Figure 7:**
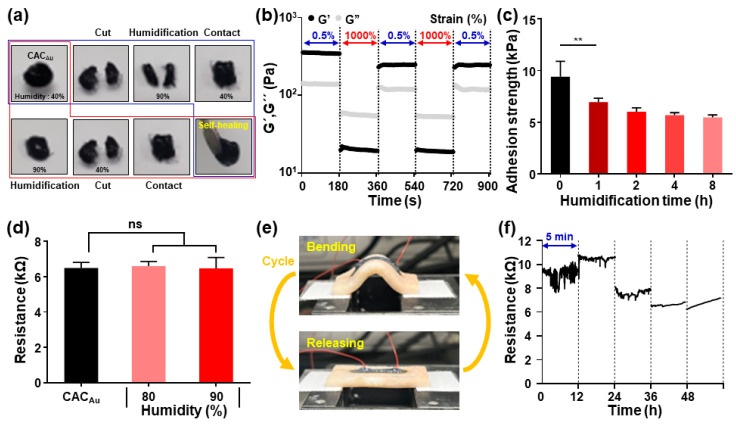
Stability of self-healing property, adhesiveness, and electrical resistance of CAC hydrogels. (**a**) Photos to show self-healing behavior of CAC_Au_ hydrogels under humidified condition (90%), and (**b**) their G′ and G″ values recovered under alternating strains of 0.5% and 1000% after 2-h incubation of the hydrogels in 90% humidity chamber. (**c**) Shear adhesion strength of the CAC_Au_ hydrogels as a function of incubation time in 90% humidity chamber. ** *p* < 0.01, one-way ANOVA. (**d**) Electrical resistance of the hydrogels under high relative humidity (80 or 90%). ns for not significant, one-way ANOVA. (**e**) Experimental settings to measure electrical resistance of the CAC_Au_ filament directly printed on porcine skin tissue during bending/releasing cycle. (**f**) Changes in the resistance of the printed filament during the cyclic deformation every 12 h (total 48 h).

The hydrogel inks for on-skin electronics should maintain their properties even under continuous exposure to high relative humidity (e.g., sweat) and repeated mechanical deformation by daily movement. Thus, we evaluated the stability of self-healing, adhesiveness, and electrical resistance of the CAC_Au_ hydrogels in high relative humidity (80 or 90%) (Figure 7a–d). They still showed self-healing behavior even after a 2 h incubation in a humid chamber (~90%) (Figure 7a), and the G′ value recovered up to 70% of its initial value (Figure 7b). Regarding their tissue adhesion, although the adhesive strength slightly decreased down to ~5 kPa under humidified conditions when compared to 9.4 ± 1.2 kPa of their initial status (e.g., before incubation in humid chamber), the value was retained for 8 h (Figure 7c). There was also no significant difference in their ionic conductivity (~6 kΩ) before/after exposure to high relative humidity (80 and 90%) (Figure 7d). Moreover, such electrical resistance was maintained even under daily deformation (e.g., repeated bending and releasing every 12 h) (Figure 7e,f). During deformation of the skin substrates with a bending–releasing cycle of 5 min every 12 h, the resistance value of the CAC_Au_ hydrogel was at a level of 6–10 kΩ, and there was no significant increase even after 48 h. All results indicate that the physical and electrical properties of the CAC hydrogels are stable enough against high humidity and continuous mechanical stress to be directly printable on the tissue.

## 3. Conclusions

We synthesized and evaluated CAC hydrogel ink for the direct printing of electronics on tissues using CMC, TA, and metal ions (to increase the conductivity). The CAC hydrogel ink exhibited excellent self-healing properties, regardless of the type of metal ion. An evaluation of the tissue adhesion of the CAC hydrogel using a UTM indicated that a sufficient tissue adhesion strength for direct printing was achieved regardless of the type of metal ion, and it was verified that the tissue adhesion was determined by the amount of metal ions. In addition, an analysis of the resistance changes under deformation from tensile strain and cyclic strain indicated that CAC_Au_ had the lowest resistance at 300% strain (8 kΩ), and the difference in the resistance due to deformation was the smallest (3 kΩ). In contrast, CAC_Fe_ had a high resistance of ≥12 kΩ at 300% strain, and a change of ≥7 kΩ in the electrical resistance occurred in the 200% strain cycle. Owing to its mechanical properties, adhesion, and electrical conductivity, the CAC_Au_ hydrogel was selected as the most suitable for direct printing. Finally, the electrical performance of electronics printed directly on tissue using the CAC hydrogel was evaluated. Electronics with various patterns printed directly on the skin tissue maintained a high resolution and uniform filament thickness and stable conductive properties under deformation due to tension, bending, and twisting. Owing to its various advantages, we expect that the CAC hydrogel will be used reliably as an ink material for the direct printing of electronics on tissue.

## 4. Materials and Methods

### 4.1. Preparation of AC and CAC Hydrogels

Sodium carboxymethyl cellulose (CMC, 700 kDa), TA, ferric chloride (FeCl_3_), gold trihydrate chloride (HAuCl_4_), and silver nitrate (AgNO_3_) were purchased from Sigma-Aldrich (Burlington, MA, USA).

An AC hydrogel was fabricated by mixing CMC and TA. The CMC solution was prepared by adding CMC to deionized distilled water (DDW) at a concentration of 5%, followed by vortexing. The AC hydrogel was prepared by mixing a TA solution (1 g/mL) with CMC solutions of different volume ratios. Each completed sample was labeled according to the volume ratios of CMC and TA.

The CAC hydrogel was prepared by mixing CMC, metal ions, and TA. The metal ion solution was prepared by dissolving HAuCl_4_ (10 wt.%), AgNO_3_ (10 wt.%), and FeCl_3_ (2.5 wt.%) in DDW. To synthesize the CAC hydrogel, 20 μL of the metal solution was added to 0.3 mL of the CMC solution, followed by mixing. Then, 80 μL of the TA solution was added, and the mixture was stirred vigorously for 5 min.

### 4.2. Rheological Characterization of AC and CAC Hydrogels

The oscillation frequency sweep test and self-healing measurements through the step strain test of the rheological properties of the AC and CAC hydrogels were conducted using a TA Instruments Discovery Hybrid Rheometer 2 (TA Instruments, (New Castle, DE, USA)). All rheological measurements were conducted using a 20-mm parallel-plate geometry with a gap size of 300 µm. The storage (G′) and loss (G″) moduli were measured at room temperature under oscillation frequency sweeps (0.1–10 Hz, at 1% strain). To demonstrate the self-healing property, G′ and G″ were measured under repeated application strains of 0.5% and 1000% for 180 s, respectively, at an osculation frequency of 1 Hz. In addition, the rheological properties of the AC-3 hydrogels (0.4 mL) after addition of urea (1, 5, and 10 M, 0.1 mL) were investigated by same oscillation frequency sweep test.

### 4.3. Tissue Adhesion Capabilities of AC and CAC Hydrogels

To evaluate the stability of CAC printing on skin tissue, the adhesion of CAC to tissue was investigated using a UTM (34SC-1, Instron, Norwood, MA, USA). First, pig skin was cut into 2 × 1 cm^2^ pieces. The CMC solution, AC, and CAC were spread between the skins, and the terminals of the substrates were pulled at a speed of 1 mm/s. The adhesion strength (kPa) was calculated by dividing the maximum load (N) by the attached area (m^2^).

### 4.4. Resistance per Strain

First, the electrical wires were fixed on an automatic one-axis stretcher (SMC-100, Jaeil Optical System (Incheon, Korea)) using double-sided tape after peeling off 0.5 cm of the sheath at one end of the wires. The hydrogel was loaded on the stretcher by approximately 2 cm using a 3-mL syringe. Finally, electrical wires and a digital multimeter (Keithley 2450 source meter, Tektronics (Seoul, Korea) were connected to the samples to measure their electrical resistances. The samples were measured in the stretched state at a rate of 3 mm/min, and for the cyclic test, the rate was 20 mm/min at strains of 0% and 200% for 100 cycles. In all cases, the initial length before the measurement was 3 mm. In addition, liquid metal (eutectic gallium indium, Alfa Aesar (Haverhill, MA, USA)) was used to maintain a stable connection between the wires and the hydrogel during measurement.

### 4.5. Direct Writing of CAC on Skin Tissue

To confirm that direct writing of electronics using CAC hydrogel is possible, CAC ink was printed on pig skin tissue using a 3D printer (Dr. INVIVO 4D, Rokit Healthcare (Seoul, Korea)). For design and printing, the “New creator K v1.57.71” program was used. The printing was conducted using a 0.6-mm nozzle and an output speed of 5 mm/s. The availability of CAC hydrogels printed in various forms, such as circles, grids, and circuits, on pig skin tissues was demonstrated using an LED test. The CAC hydrogel transformed the printed skin tissue by stretching, bending, twisting, and wetting in PBS, and the LED emission was confirmed.

### 4.6. In Vitro Cytotoxicity Test

To evaluate cytotoxicity of the CAC hydrogels, mouse fibroblast cells (L929) were pre-cultured in Dulbecco Modified Eagle Medium (DMEM supplemented with 10% fetal bovine serum and 1% penicillin/streptomycin). To collect the eluates of the CAC_Au_ inks, 40 mg of the inks was incubated in cell culture media (10 mL) for 24 h. The cells were seeded in a 48-well plate (2 × 10⁴ cells per well) and cultured in a 5% humidified CO_2_ incubator at 37 °C. After culturing overnight and washing with Dulbecco’s Phosphate-Buffered Saline (DPBS), the media containing the sample eluates was supplemented in the well. The cell viability at 24 and 48 h was evaluated using Live/Dead assay kit (Thermo Fisher Sci. (Seoul, Korea)) The live cells were stained with Calcein AM solution (2 μM) and dead cells were stained with Ethidium homodimer-1 solution (4 μM) (0.2 mL of total working solution), and then incubated for 1 h at 37 °C. Finally, the live/dead cells were observed using fluorescence microscopy (DMi8, Leica (Wetzlar, Germany)). The number of either green dots for live cells or red dots for dead cells was counted using Image J software, and cell viability (%) was calculated as the ratio of the number of live cells to the total number of cells.

### 4.7. The Stability Characterization of CAC Hydrogel in High Relative Humidity Conditions

Self-healing property, tissue adhesiveness, and ionic conductivity of CAC hydrogels were evaluated in high humidity condition (80–90%). The humid chamber equipped with a hygrometer was manually prepared using acrylic plates. First, the self-healing property of CAC_Au_ hydrogel was confirmed after cutting and 2-h incubation of the hydrogel pieces in the chamber. In addition, G′ and G″ values of the hydrogels after 24-h incubation in the chamber were monitored using oscillatory rheometer (at 1 Hz, each step for 180 s under alternating strain between 0.5% and 1000%). Second, tissue adhesiveness of the hydrogels as a function of incubation time in the humid chamber was measured in a same manner with Section 4.3. Finally, the ionic conductivity of the hydrogels was also measured without strain in the same manner as in Section 4.4 as a function of incubation time under humidity conditions of 80% and 90%.

### 4.8. The Long-Term Stability of Electrical Resistance of CAC Hydrogels

To evaluate the stability of electrical resistance of the CAC hydrogel against long-term repeated tissue deformation, the single filament (length = 2 cm, 18-gauge needle) was printed using CAC_Au_ hydrogel on the porcine skin. The substrate tissue was fixed on the one-axis stretcher using double-sided tape. Electrical wires and a digital multimeter were connected to the samples to measure their electrical resistance in between two terminals of the printed filament. For the cyclic test, the sample was bent at a rate of 20 mm/min with 1 cm for 5 cycles and the measurement was conducted every 12 h.

## Figures and Tables

**Table 1:** Precursor compositions of different AC hydrogels.

	CMC (mg)	TA (mg)	DDW (mL)
CMC_0.5_	25	0	1
AC-1	25	500	1
AC-2	33.2	333	1
AC-3	37.5	250	1

**Table 2:** Precursor compositions of different CAC hydrogels.

	CMC (mg)	TA (mg)	Metal Ion (mg)	DDW (mL)
CAC_Au_	37.5	250	Au/5	1
CAC_Ag_	37.5	200	Ag/5	1
CAC_Fe_	37.5	250	Fe/1	1
CAC_Au_@5	37.5	250	Au/1.25	1
CAC_Au_@10	37.5	250	Au/2.5	1

## Data Availability

The data presented in this study are available in the article.

## Supplementary Materials

The following supporting information can be downloaded at: https://www.mdpi.com/article/10.3390/gels8060336/s1, Figure S1. Evaluation and comparison of storage modulus (G′) and loss modulus (G″) due to hy-drogen bond collapse between CMC and TA due to disruption of hydrogen bond by various con-centration of urea; Figure S2. In vitro cytocompatibility of CACAu. (a) The fluorescent images of L929 cells at 24 and 48 hours after treatment of the CACAu relesates as a function of concentration (0, 0.5, 1, and 2 mg/mL). (b) Quantitative analysis of the cell viability. All data are expressed as mean ± s.d. One-way ANOVA, **** *p* < 0.0001, and ns for not significant; Movie S1. The stability of the on-tissue printed filaments upon soaking in PBS and against physi-cal deformation of porcine skin.
