# Supplementary material for: Tissue Adhesive, Conductive, and Injectable Cellulose Hydrogel Ink for On-Skin Direct Writing of Electronics
Source: Gels. 2022 May 30;8(6):336. doi: 10.3390/gels8060336 (PMC9222510; doi:10.3390/gels8060336)
Supplement: Supplementary file 1 [file gels-08-00336-s001.zip › gels-1719444-supplementary.pdf]

gels

MDPI

Article

Tissue-Adhesive, Conductive, and Injectable Cellulose Hydrogel Ink for On-Skin Direct Writing of Electronics

Subin Jin [1], Yewon Kim [2], Donghee Son [2,3,4,*] and Mikyung Shin [1,4,5,*]

1 Department of Intelligent Precision Healthcare Convergence, Sungkyunkwan University (SKKU), Suwon 16419, Korea; subinjin@g.skku.edu
2 Department of Electrical and Computer Engineering, Sungkyunkwan University (SKKU), Suwon 16419, Korea; ywkim0726@gmail.com
3 Department of Superintelligence Engineering, Sungkyunkwan University (SKKU), Suwon 16419, Korea
4 Center for Neuroscience Imaging Research, Institute for Basic Science (IBS), Suwon 16419, Korea
5 Department of Biomedical Engineering, Sungkyunkwan University (SKKU), Suwon 16419, Korea
* Correspondence: daniel3600@g.skku.edu (D.S.); mikyungshin@g.skku.edu (M.S.)

Supplementary Materials

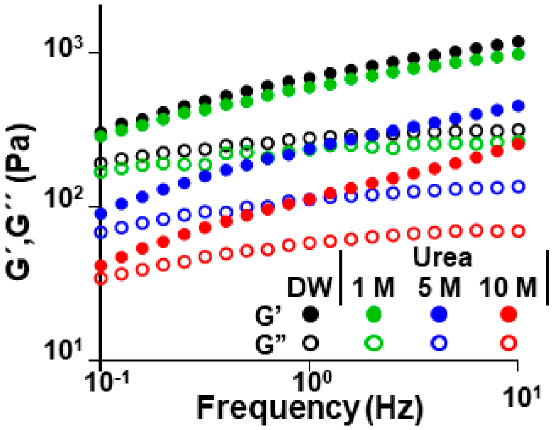

Figure S1. Evaluation and comparison of storage modulus (G′) and loss modulus (G′′) due to hydrogen bond collapse between CMC and TA due to disruption of hydrogen bond by various concentration of urea.

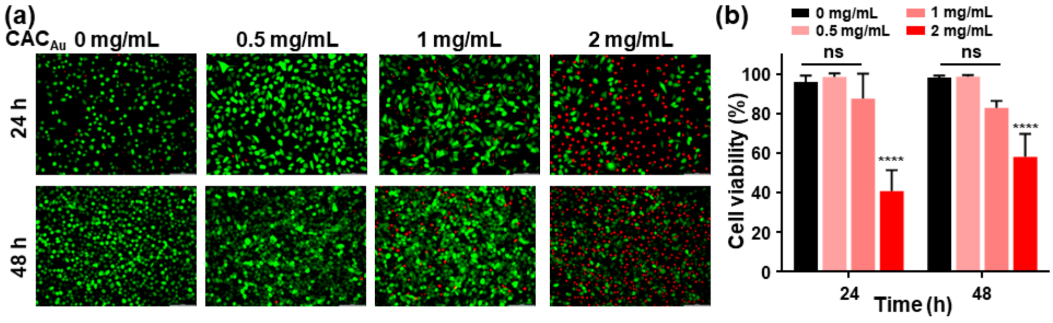

Figure S2. In vitro cytocompatibility of CAC$_{Au}$. (**a**) The fluorescent images of L929 cells at 24 and 48 hours after treatment of the CAC$_{Au}$ relesates as a function of concentration (0, 0.5, 1, and 2 mg/mL). (**b**) Quantitative analysis of the cell viability. All data are expressed as mean ± s.d. One-way ANOVA, ****$p < 0.0001$, and ns for not significant.

Movie S1. The stability of the on-tissue printed filaments upon soaking in PBS and against physical deformation of porcine skin.
